# Two decades of the CHNRI method (2006–2025): Tracking its evolution and contribution to the emerging field of ideometrics

**DOI:** 10.7189/jogh.15.01006

**Published:** 2025-10-01

**Authors:** Igor Rudan

**Affiliations:** 1 Centre for Global Health, Usher Institute, The University of Edinburgh, Edinburgh, UK; 2 Nuffield Department of Primary Care Health Sciences, Oxford University, UK; 3 Green Templeton College, Oxford University, UK

## Abstract

This paper tracks the evolution of the Child Health and Nutrition Research Initiative (CHNRI) method for setting health research priorities and situates it within a much broader, emerging field of ‘ideometrics’ – the quantitative study of how ideas can be generated, evaluated, and prioritised. First presented in 2006, the CHNRI method tackled three key barriers to research priority setting: an infinite number of possible research ideas, uncertainty about future payoffs of investing in research, and the need for a fair, transparent, legitimate, and broadly acceptable consensus. Its proposed solutions were based on the systematic nature of idea generation, explicit context framing, transparent criteria, and expert crowdsourcing, while its scores reflected ‘collective optimism’ towards many ideas that could be optionally weighted by funders and stakeholders. Early demonstrations of its usefulness were followed by the establishment of a landmark World Health Organization (WHO) programme that set priorities across the leading causes of global child mortality. The resulting publications catalysed adoption of the method by major agencies and many national governments. Within a decade, the CHNRI method became the most widely used approach to health research priority setting. The review of the first 50 exercises revealed its practical advantages: its systematic scope, transparency, inclusiveness, flexibility, simplicity, low cost, and publishable outputs. Its ‘natural evolution’ within the global health research community led most users to sensibly adapt its standard criteria to their specific contexts. Experiments on quantitative properties of human collective knowledge and opinion demonstrated accuracy within domains of expertise. They also showed that saturation of experts’ collective opinion occurs with 45–55 scorers, achieving very stable rankings. Subsequent advances introduced bootstrapped confidence intervals, an information-theory expert agreement metric, and clustering analysis to detect scorer sub-structures, strengthening the method. Consultations with funders clarified ‘funding attractiveness’ as a complementary criterion, improving the method’s policy traction. By the year 2025, the CHNRI method underpinned major exercises led by the leading international organisations in all of the world’s regions, and supported research prioritisation in many challenging national and regional settings. A pivotal recent shift is the integration of artificial intelligence (AI)-based large language models: the CHNRI method can now accommodate AI as a partner in all steps of the priority-setting process. Moreover, years of CHNRI practice motivated a broader theoretical move: viewing the brain’s ‘perception of ideas’ as an underappreciated human sense. These advances call for a more quantitative, testable, and replicable future developments in which the CHNRI method will contribute to ‘ideometrics’ – an emerging scientific field devoted to generating, evaluating and prioritising ideas that are likely to lead to human progress in health and beyond.

This editorial summarises the development, implementation and evaluation of the Child Health and Nutrition Research Initiative (CHNRI) method and its contribution to the emerging field of ‘ideometrics’. The CHNRI method is a structured process that can be used to systematically generate, evaluate and prioritise research ideas. It assists research priority setting at all levels – global, regional, national and local [1,2]. Historically, it found its first applications in child health and nutrition research, but soon expanded to many other areas of health research. It explicitly considers the context in which research ideas are prioritised, while being transparent about the criteria that were used in its process [3–6]. The standard elements of this context include ‘space’ (*i.e.* geographic boundaries), ‘time’ (*i.e.* when are the returns on investments expected), ‘population’ (*e.g.* age groups, sex, special populations), ‘target’ (*i.e.* burden of all or some diseases), and ‘investment style’ (*i.e.* low, moderate, or high-risk), but further descriptors of the context can also be included. Its standard criteria follow the progress of health research, from study design and knowledge generation to translation and implementation. They thus include the likelihood of ‘answerability/feasibility’, ‘effectiveness’, ‘deliverability/affordability/sustainability’, ‘maximum potential to reduce disease burden’ and ‘impact on equity’. However, many other criteria can also be flexibly used, depending on the context, while some of the standard ones can be dropped if they are unlikely to discriminate well between the many proposed research ideas.

The CHNRI method engages a diversity of stakeholders – funders [7], researchers [8], and policymakers [9]. It introduced several key conceptual advances [10] and contributed to understanding quantitative properties of human collective knowledge and opinion [11,12]. Based on the relatively recent concept of crowdsourcing [13,14], it is well suited to addressing the needs of the poor and underprivileged in the world, setting research priorities in a democratic, transparent, and simple way [15]. Within the first ten years since its introduction, it became the most widely used method for prioritising health research globally [16].

Today, twenty years after its introduction, the CHNRI method has established itself as an important innovation within the emerging field of ‘ideometrics’ – the science of generating, evaluating, and prioritising ideas. ‘Ideometrics’ includes dozens of diverse methods and approaches that have historically been used to assist those three interconnected tasks in many areas of human activity. Once exclusively a human feature, ideas can now also be generated by artificial intelligence (AI) models, with the CHNRI method being the first within the field of ideometrics to recognise and accommodate this major technological progress [17,18].

## YEARS 2006–2008

### The development of the CHNRI method

At the 10th Global Forum for Health Research held in Cairo, Egypt, in 2006, a new method for setting health research priorities was introduced. I led the transdisciplinary work on its development in the preceding two years (2005–06) as the consultant of the CHNRI of the Global Forum for Health Research in Geneva, Switzerland, which is why it became known as the CHNRI method [1]. This work was funded by the World Bank, which enabled the participation and input from a transdisciplinary group of 26 experts, whom I invited to different stages of the development process. I was continuously supported in this work by the two world-renowned global health experts who coordinated the CHNRI: Robert E. Black from the Johns Hopkins University in Baltimore, USA, and Shams El Arifeen from the International Centre for Diarrhoeal Disease Research, Bangladesh (iccdr,b).

The experts provided feedback on my progress from their disciplinary perspectives at the meetings in Geneva, Switzerland; Baltimore, USA; Dubrovnik, Croatia; and Mumbai, India in 2005, and then in Cape Town, South Africa; Toronto, Canada; and finally in Cairo, Egypt, in 2006. Interestingly, the term ‘crowdsourcing’ was first coined in the magazine ‘Wired’ the very same year (2006) [19] and has described well the core innovative component of the CHNRI method. It reflected an emerging opportunity to use human collective knowledge and opinion as a powerful tool in various fields. The advent of the World Wide Web and e-mail, which were becoming widespread in the previous years, enabled invitation to participation and collection of responses from many people in ‘real time’ in a digital form.

After the previous approaches were reviewed [3], and the stakeholders required for the process were carefully mapped and their roles in the process considered and assigned [4], the CHNRI method then identified and proposed to solve several seemingly intractable challenges in prioritising health research [5]. These difficult challenges included, for example, the infinity of possible research ideas; then, the unpredictability of the future and the handling the uncertainty of the possible outcomes of research; and finally, the reaching of an consensus acceptable to all stakeholders, *i.e.* the consensus over the outcomes in cases where one’s own favourite ideas did not come out on top.

Although its development required a transdisciplinary group of experts to propose some feasible solutions to such challenging problems, the final version of the CHNRI method was simple. In the first step, it defined the existing context within a chosen field of health research and identified a small set of criteria that would recognise one research idea as being ‘better’ than another. These criteria could have been numerous, but the ‘template’ of five of them chosen for the unmodified CHNRI method included: the likelihood that the proposed research idea would be answerable; the likelihood that it would lead to an effective intervention; the likely deliverability, affordability, and sustainability of the future intervention; the likely maximum reduction of the existing disease burden; and any likely effects on equity. In the second step, the method resorted to crowdsourcing: dozens of the leading experts in the chosen field of health research would be invited to propose several of their best research ideas. Then, a ‘consolidated’ list of the research ideas could be developed after similar ones were merged and duplicates were removed. In the third step, all the experts would ‘score’ the ideas by assessing their likelihood of satisfying each criterion. They would do so by answering simply ‘yes’ or ‘no’, while occasionally being allowed to use an ‘informed maybe’ or simply leave the scoring field blank if they did not have enough knowledge to make this judgement [6]. As a result, the collective opinion of dozens of leading experts in the field of health research about their own 100–200 research ideas would be captured and visualised through this expert sourcing.

The final result of the CHNRI process is a simple table that measures the ‘collective optimism’ of the leading experts towards each research idea in terms of how it would satisfy each of the criteria. All the resulting numerical scores are intuitive, as they range between 0–100%. Then, the overall research priority score (RPS) can be calculated as a simple average of all criterion-specific scores. Moreover, specific weights can be placed on some criteria that are seen as more important by the funders or wider external stakeholders, allowing funders, stakeholders, patients, or even members of the public to influence the overall scores [6].

This process is quick and cheap, as well as systematic, flexible, and easy to tailor to many different needs. Also, it is democratic, transparent, and replicable. Its outputs address the needs of the funders of health research; upon the completion of each ‘CHNRI exercise’, the funders can easily see what do many leaders within a chosen research community collectively think about many different research ideas, which they themselves nominated as the most promising. Importantly, the final rankings are influenced by everyone equally; each expert’s input independently contributes only a very small fraction to the overall result, so that no single person can have undue influence on the final ranking list. The final scores across all the criteria typically range from just above 90% to below 20%, suggesting that the CHNRI method performs well at discriminating between the many competing research ideas.

The four foundational papers on the CHNRI method were published in the Croatian Medical Journal in 2007 and 2008 [3–6]. This journal was chosen because it was among the few at the time to be fully open-access; it did not require any article processing or usage fees and its content to was freely reusable. This seemed an appropriate way to publish a methodology that was intended to assist everyone in need of such a tool, as those in greatest need were, primarily, the populations of low- and middle-income countries (LMICs). Addressing their main health challenges would improve equity in international research [20] and reduce the 10/90 gap identified by the Global Forum for Health Research, where less than 10% of global health research resources were being spent on conditions that affect 90% of the world’s population [21].

### First implementations of the CHNRI method

The first publication that drew the attention of the international research community to the new method was a policy paper entitled ‘Childhood Pneumonia and Diarrhoea: Setting our Priorities Right’, published in ‘The Lancet Infectious Diseases’ in 2007 [22]. This paper closely followed the important insight published previously by the Child Health Epidemiology Reference Group (CHERG) of the World Health Organization (WHO) and the United Nations Children’s Fund (UNICEF), to which I also contributed. The CHERG experts identified childhood pneumonia and diarrhoea as the leading causes of child deaths globally, that were jointly responsible for nearly half of child mortality. The CHNRI method was suggested as a possible tool that could assist governments and international organisations in directing research funding towards achieving the largest gains in mortality reduction.

The first country to quickly respond to this publication and the opportunity to utilise the new method was South Africa, where the first actual implementation of the CHNRI method took place. I travelled to Cape Town, trained a group of prominent local researchers in using the method, and assisted in the conduct of the exercise based on the originally proposed set of the CHNRI criteria. Led by Tomlinson and colleagues, 63 proposed research ideas were scored by a group of six leading national child health experts, resulting in a paper entitled ‘Setting Priorities in Child Health Research Investments for South Africa’, published in the journal ‘PLoS Medicine’ in 2007 [23]. It was the first proof of concept that the methodology can be successfully completed and useful to funders and policymakers.

Two other groups decided to become early adopters and use the CHNRI methodology within their own fields. Chisholm and colleagues were preparing an influential piece on the challenges with health services for mental disorders. Within their paper, they conducted a CHNRI exercise to set research priorities, involving 24 expert scorers who proposed 100 research ideas, while keeping the original set of the proposed CHNRI criteria. This paper was published in ‘The Lancet’ in 2007 [24].

Soon thereafter, Walley and colleagues were working on another influential paper, addressing challenges in global primary health care. Within their paper, they also decided to conduct a CHNRI exercise, involving 20 expert scorers and 69 proposed research ideas. This was the first innovative application of the CHNRI method, where the original set of criteria was modified according to the needs of the exercise: the criterion ‘likelihood of effectiveness’ was replaced by ‘feasibility of undertaking’, while an entirely new, sixth criterion was added: ‘likelihood to fill a critical gap in knowledge’. The paper, published in ‘The Lancet’ in 2008 [25], represents the first independent modification of the CHNRI method by users.

### Presentation of the CHNRI method at the 9th, 10th, 11th, 12th and 13th Global Forum for Health Research

One of the first meetings of the team involved in the development of the CHNRI method was held at the 9th Global Forum for Health Research in Mumbai, India, in 2005, where I outlined and presented the key challenges to the audience. Then, I first presented the CHNRI method, and distributed the first published version [1], at the 10th Global Forum for Health Research in Cairo, Egypt, in 2006. Over the next two years, the method’s four foundational papers were published in an open-access journal, making it free for everyone to use [3–6]. I presented this progress at the 11th Global Forum for Health Research in Beijing, China, in 2007. The first examples of its implementation and relevance to policymakers reached very broad audiences through the publications in ‘The Lancet’, ‘The Lancet Infectious Diseases’, and ‘PLoS Medicine’ journals, serving as early platforms for the wide dissemination of the method’s utility. Following these advances, two more presentations with additional updates on the CHNRI method’s development and implementation were given at the 12th Global Forum for Health Research in Bamako, Mali, in 2008, and then in Havana, Cuba, in 2009. The time was right for a major international organisation to consider addressing its needs through the application of the CHNRI method.

## YEAR 2009

### The WHO sets research priorities to address global child mortality using the CHNRI method

In 2009, the Department of Maternal, Newborn, Child, and Adolescent Health of the WHO decided to use the CHNRI method to conduct five separate exercises, addressing the five most important causes of global child mortality based on the CHERG’s technical work. I was again engaged as the consultant who coordinated the process. It resulted in five landmark publications that directed global funding for priority research to address the five key causes of global child mortality, which was the 4th Millennium Development Goal of the United Nations. The output included papers by Fontaine and colleagues on childhood diarrhoea [26], Bahl and colleagues on neonatal infections [27] and preterm birth and low birth weight [28], and Lawn and colleagues on birth asphyxia [29], while I led the work on childhood pneumonia [30].

These five papers, published mainly in the journal ‘PLoS Medicine’, raised considerable international interest among funders and policymakers alike. The novelty was that all five papers largely promoted one specific domain of research called ‘health policy and systems research’. Given the urgency of prioritising research for reducing a burning problem of global child mortality, it is not surprising that the investments in this type of health research came on top. Dozens of international experts invited by the WHO to take part in these CHNRI exercises converged towards the suggestion that lot more impact could be achieved with the resources, interventions and knowledge that we already have as a research community, but they are not being used efficiently. The number of research ideas typically addressed in these exercises ranged from 61 to 156, while the number of scorers ranged from 13 to 45.

In the meantime, Tomlinson, who led the first national-level exercise in South Africa, mobilised the WHO staff within his own fields of research interest, resulting in two additional high-profile publications with the WHO participation: a CHNRI exercise on disabilities, published in by ‘The Lancet’ in 2009 [31], and research priorities for global mental health, published by the ‘Bulletin of the World Health Organization’ in 2009 [32]. These two papers are important because they continued to demonstrate, following the papers by Chisholm and colleagues [24] and Walley and colleagues [25], that the CHNRI method is very flexible and can be used to set research priorities in different fields of health research. Another notable implementation of the CHNRI method in 2009 is the one by Brown and colleagues, who focussed on research priorities in zinc supplementation for children in low- and middle-income countries [33].

### The WHO convenes a meeting with funders

Having completed the five CHNRI-based exercises, the WHO was keen to engage funders and share the results with them to ensure their real-world impact. I was also interested in whether it would be possible to add yet another criterion to the CHNRI exercises – ‘attractiveness to funders’ – and what would be its basis. In 2009, together with the representatives of the WHO Maternal, Newborn, Child, and Adolescent Health (MNCAH), I coordinated a meeting at the WHO in Geneva, Switzerland, which gathered 40 representatives from funding organisations, including the Bill and Melinda Gates Foundation (BMGF); the Wellcome Trust; the USA National Institutes of Health (NIH); the UK Department for International Development (DFID); Save the Children; the International Clinical Epidemiology Network (INCLEN); EPICENTRE; the UNICEF; the United States Agency for International Development (USAID); PATH; the Ministry of Science and Technology of India; the ministries of health of Zambia, Pakistan, and Brazil; the Global Forum for Health Research, Trinity Global Support Foundation, Children's Investment Fund Foundation (CIFF), and Osaka Research Institute for Maternal and Child Health. Sixteen representatives of funding agencies agreed to participate in the meeting on condition of anonymity. Their views did not represent official agency positions and implied no funding obligations [7].

After the aims of the consultation were explained, participants were presented with the top 10 research priorities for each of the five major causes of child deaths – pneumonia, diarrhoea, birth asphyxia, neonatal infections, and preterm birth/low birth weight – identified through WHO-coordinated CHNRI exercises [26–30]. These 50 priorities represented the top 5% of all submitted research ideas. The WHO coordinators explained each priority before participants independently assessed them. Funding attractiveness was measured in two ways. First, participants ranked the priorities by likelihood of funding under current agency policies. Second, they distributed a hypothetical USD 100 among those most fundable, where they assigned ranks from 1 (most likely to be funded) to 10 (least likely) and allocated money accordingly. Sixteen completed scoring sheets showed that average ranks ranged from 3.7 to 7.2, and allocated amounts from USD 2.5 to USD 20.1, with broad consistency between rankings and dollar values. This WHO MNCAH-based consultation highlighted that funders’ perspectives on research attractiveness often differed from those of researchers and stakeholders, as well as across types of funding organisations [7].

The meeting reinforced the importance of involving funders early in exercises like the CHNRI to build ownership of results. The criterion of ‘attractiveness to funders’ should complement those valued by researchers and other stakeholders in the CHNRI framework; otherwise, the prioritisation outcomes may have less influence on investment decisions. The CHNRI method offers funders transparent comparisons of risks and benefits across competing research ideas, translating the research community’s collective judgment into an accessible and replicable format. However, challenges remain in ensuring funders’ systematic engagement, clear communication of results, and commitment to using CHNRI outputs. The conclusion from this meeting was that developing tools to track the CHNRI’s influence on funding decisions and detect shifts in investment priorities, especially where public funds are involved and the main goal is cost-effective health improvement, will remain an even greater challenge [7].

## YEAR 2010

### The BMGF sets research priorities to accelerate emerging interventions

The WHO series on priority research for reducing child mortality and the subsequent meeting with the funders attracted interest from another major funder of global health research – the BMGF – which at the time funded many large initiatives and projects to reduce global child mortality. I received two major grants from the BMGF to modify the CHNRI method to focus only on emerging interventions based on innovative technological solutions that could accelerate implementable and feasible interventions. The target diseases for these grants were any novel interventions for childhood pneumonia and diarrhoea, and new diagnostic solutions for neonatal infections.

This work resulted in a series of papers published as a collection in the journal ‘BMC Public Health’. They were based on a method that had nine criteria, where ‘low development cost’, ‘sustainability of implementation’, ‘acceptability to health care providers’ and ‘acceptability to end users’ were added to the standard five criteria. The series addressed the emerging interventions against pneumococcus [34], staphylococcus [35], meningococcus [36], respiratory syncytial virus [37], measles [38], influenza [39], oxygen systems [40], and diarrhoea [41]. Later, an integrative paper addressing the potential role of emerging diagnostics to prevent morbidity and mortality from newborn infections was published, introducing a new tool for assessing the impact – ‘Pathways to Survival’ tool (PATHS) [42].

## YEAR 2011

### Presentation of the CHNRI method at the 19th World Congress of Epidemiology in Edinburgh, UK

In 2011, the 19th International Epidemiological Association’s World Congress of Epidemiology was held in Edinburgh, UK, where I was given the privilege of delivering a plenary presentation on the CHNRI method’s development and implementation over the first five years of its existence (2007–11). My presentation was later published in the journal ‘Public Health’, with the title ‘Global health research priorities: Mobilising the developing world’ [15]. The presentation further popularised the CHNRI method and made many public health experts internationally, and especially in LMICs, aware of its potential.

### Multiple other implementations and international uptake

As a result of the increasing visibility of the CHNRI-based priority setting exercises, some global health experts published notable further examples of their use in some of the most high-profile journals. Three were published in the journal ‘PLoS Medicine’: Tol and colleagues set research priorities for psychosocial support in humanitarian settings, where 74 research ideas were scored by 82 experts [43]; George and colleagues set implementation research priorities for stillbirths and preterm births based on 59 research ideas and 29 scorers [44]; and Lienhardt and colleagues set research priorities to tackle tuberculosis, with 50 experts scoring 250 research ideas [45]. In the meantime, Flenady and colleagues set research priorities to address stillbirths as a global health issue, looking at 279 research ideas with 50 expert scorers, and published their work in ‘The Lancet’ [46]. Finally, Collins and colleagues used a modified CHNRI process to identify research priorities for impaired mental health among 164 research ideas assessed by 33 expert scorers, publishing their work in the journal ‘Nature’ [47]. At this point, the CHNRI method was truly established and widely applied, and its applications were finding their way to the most prestigious and widely read scientific journals.

## YEARS 2012–2015

The following years saw the widespread use of the CHNRI method at national, regional, and global levels, as well as its first implementations outside of the field of health research, showing it to be flexible and able to universally address various challenges.

### Further examples of global-level implementation

Notable papers from these four years that prioritised ideas in various fields of health research using the CHNRI method included those by Hindin and colleagues, who focussed on prioritising adolescent sexual and reproductive health needs [48]; then, Dean and colleagues, on preconception care [49]; Wazny and colleagues, who revised and improved the WHO’s original exercise on childhood diarrhoea [50]; Yoshida and colleagues who addressed global newborn health [51]; Souza and colleagues, who prioritised research for maternal and perinatal health [52]; Ali and colleagues, on unmet needs in family planning [53]; Morof and colleagues, on improving neonatal survival in humanitarian emergencies [54]; Tomlinson and colleagues, on intellectual disabilities and autism [55]; Wazny and colleagues, on integrated community case management [56]; Campbell and colleagues, on family planning [57]; Angood and colleagues, on acute malnutrition in infants [58]; and Velayutham and colleagues, on drug resistant tuberculosis [59]. Importantly, nearly all of these exercises were being initiated independently of the group that developed the CHNRI method and without the need for consultancy support from the originators, implying that the research community was increasingly adopting the method and finding it simple enough to use for many different needs and in various contexts. The exercises were also becoming increasingly large and sophisticated, involving an ever-larger number of experts and, consequently, scoring an increasing number of questions in each exercise.

### Further examples of regional- and national-level implementation

An example of a regionally focussed exercise conducted in this period was the work by Rollins and colleagues in three African countries – Malawi, Nigeria and Zimbabwe – who mobilised 191 scorers to prioritise 90 implementation research ideas on to prevent mother-to-child transmission of HIV [60].

The examples of national-level implementation in this period were the papers by Sekar and colleagues prioritised research to address zoonotic diseases in India [61]; Gregorio and colleagues, who focussed on impaired mental health in Brazil [62]; and Arbour and colleagues, who set priorities for research that could assist children at developmental risk in Chile [63].

It is also worth noting that, in this period, the CHNRI method also entered the scientific literature in the Chinese language, as two exercises were performed in China [64,65]. One of them, conducted by Li and colleagues and published in the journal ‘Chinese Health Services Management’ in 2014, focussed on maternal and child health services and it had the reduction of morbidity and mortality among mothers and children as its focus [64]. The other one, by Yang and colleagues, published in the ‘Chinese Journal of Health Policy’ in 2015, addressed health policy research direction for all-cause morbidity and mortality [65].

### The first use of the CHNRI method outside health research

In this period, the first papers began to appear that applied the CHNRI method outside the field of health research altogether. The first such example was the paper by Read and colleagues, who applied the approach in the remarkably important field of education in LMICs. This paper was published in the journal ‘Current Issues in Comparative Education’, with 89 proposed ideas scored by 37 scorers [66].

## YEAR 2016

### The first PhD students advancing the CHNRI method

The CHNRI method’s uptake and implementation attracted the first PhD students to the University of Edinburgh and the London School of Hygiene and Tropical Medicine. They were keen to further improve the aspects of this methodology. Sachiyo Yoshida from the WHO set out to develop better answers to the typical reviewers’ comments on the CHNRI-based articles. She sought whether several common criticisms hold true: that the list of research ideas is not exhaustive enough; that another group of experts would produce substantially different final rankings; and that there must be a sub-structure within the identified expert group that introduces biases to the rankings in an unpredictable way. She re-analysed the results from several previously conducted CHNRI exercises and, through a series of experiments and statistical modelling, provided several potentially novel and useful insights on quantitative properties of collective human knowledge [11] and opinion [12].

Yoshida and I, joined by her other supervisor Simon Cousens, first asked a larger group of about 200 persons with expertise in one specific area to answer questions that have a precise numerical value as a correct answer. We asked them to first answer ten questions in their own area of expertise, then ten more questions where they were likely to have some (general) knowledge, and finally, ten questions from an entirely different area of expertise where they were not expected to have any knowledge. Our experiments showed that the collective knowledge of a group with expertise in one specific area should always be very close to the true answer. In most cases, and under most assumptions, the average answers from the collective will be more accurate over the ten questions than the results of an ‘average’ individual in the group. Moreover, the accuracy of collective prediction may be enhanced by allowing the individuals with low confidence in their answer to withdraw from answering, which further improves the collective prediction. However, the average collective answers were less accurate when they concerned general knowledge questions, and entirely inaccurate when they dealt with questions in another area of highly specific expertise, which they themselves did not have [11].

These experiments provided strong support to both ‘crowdsourcing’ as a robust approach for assessing the collective optimism of a group of experts towards the value of research ideas, and also for relying on experts only, while finding other roles for the funders and different stakeholders. They demonstrated that collective knowledge is most valuable when it is drawn from the experts in a field, while including any other group will reduce the accuracy of the collective prediction [11]. Moreover, the scoring system that allows withdrawal from responding when one has insufficient knowledge to contribute also gives more accurate predictions, confirming that the CHNRI method has been using the right approaches since its introduction. It was always focussed on maximising the value of collected information from the experts through crowdsourcing, based on relevance, credibility, and leverage of the collected information, as I explained in my later work on the ‘value of information’ concept [67] - and this made it useful.

Furthermore, we observed that for each new invited expert, the likelihood of contributing an idea that hasn't already been nominated would inevitably start to decrease. This implied that there must be a point of a near-saturation of proposed research ideas. This point will be reached more quickly in smaller research fields, while in large and broad fields it may take several hundreds of invited experts, each providing several new ideas. Our observation implies that although the pool of possible research ideas might indeed be infinite, in reality, it is possible to achieve a comprehensive representation of the spectrum of the feasible research ideas within a well-defined context through a large enough sample of invited experts.

Furthermore, statistical simulations that used a gradually increasing sample of experts from completed exercises showed that there is also a point – usually when about 45-55 researchers are included in the process of scoring – after which it is no longer possible to get a statistically significantly different ranking of research ideas. This holds true even if hundreds of scoring experts are added to the scoring process, provided that they are all knowledgeable enough about the subject. Interestingly, this finding suggested that human collective opinion saturates and stabilises quite quickly, requiring only 45–55 experts with sufficient knowledge of the subject [12] – an insight recently replicated by an entirely independent group in their own priority-setting exercise [68].

Adding to this, Wazny and colleagues focussed on crowdsourcing as the core component of the CHNRI method. They first reviewed the applications of crowdsourcing in all fields [13] and in the field of health [14]. Then, they systematically assessed its potential uses in addressing problems in global health, conflict and humanitarian settings. They used the CHNRI method to generate a ranked list of potential uses of crowdsourcing in global health and in conflicts, as a particularly innovative application of the method [69]. They engaged 94 experts in global health and crowdsourcing who scored 239 ideas. Most of those proposed research options were related to problem solving (112 ideas) or data generation (91 ideas). The top ranked ideas involved using healthcare workers to crowdsource information about disease outbreaks, using crowdsourcing for vital registration and for improving maternal and child health. Wazny and colleagues also noted that many ideas reflected Sustainable Development Goals (SDGs), so she proposed that crowdsourcing may be an innovative solution to achieving some of the SDGs [69]. Another one of Wazny and colleagues’ major contributions to the development of the CHNRI method was setting weights for 15 CHNRI criteria at the global and regional level using public stakeholders, through an Amazon Mechanical Turk study [70].

### Journal of Global Health’s series of seven papers advancing the CHNRI method

Ten years after its first presentation, in 2016, the CHNRI method had already become the most widely used for setting health research priorities in the 21st century, surpassing the Delphi technique and other approaches [16]. Building on the work of the first PhD students interested in advancing the method, the time was right to revise and improve guidelines for various elements of the method and to reflect on what was learned from its implementation over the first decade. The strategies for engaging funders [7], researchers [8], and other stakeholders [9] became clearer based on the gained experience, so the instructions on those three important steps of the CHNRI exercise were laid out for the prospective users [7-9]. Likewise, numerous interactions with the users exposed the most frequent questions about the CHNRI method’s main features, so the key conceptual advances were further elaborated to facilitate new exercises and encourage uptake in many additional settings [10]. The experiments in understanding quantitative properties of human collective knowledge [11] and human collective opinion [12] were also added to this series, as novel and original contributions enabled by the CHNRI method. Finally, a review of the first 50 publications that described the implementation of the CHNRI method was conducted and published as the last paper of this series (see below) [71].

### The first review of the CHNRI-based exercises – understanding the natural evolution of the CHNRI method

The first 50 CHNRI priority-setting exercises, published between 2007 and 2016, reached out to nearly 5000 researchers, policymakers, and programme officers, seeking their participation in the generation of research ideas and their scoring by the agreed criteria. The initial response rate across all exercises was above 60%, with more than 3000 experts submitting about 10 000 ideas (more than three per expert). Eventually, 4282 ideas were scored (an average of 86 per exercise), indicating that more than 50% of the initially submitted questions were duplicates/redundant and merged with other questions, or poorly framed and discarded. Eventually, 2403 invited experts participated in scoring (an average of 48 per exercise). The journals most receptive to the first 50 papers were ‘PLoS Medicine’ (n = 10), ‘BMC Public Health’ (n = 7) and ‘The Lancet’ (n = 6) [71].

Many of the exercises addressed child morbidity or mortality and improved development (n = 23), maternal, perinatal, and sexual health (n = 4), and several major infectious diseases that are not primarily maternal, newborn, and child health issues (n = 3). The most frequent application outside of this initial focus was mental health (n = 8), and all-cause mortality, morbidity, and disability in adults (n = 4). Most exercises focussed on LMICs (n = 25), while others had a global (n = 16), national (n = 7), and sub-national (n = 1) scope. Twenty-eight papers focussed on children and/or newborns, eight on adolescents and young adults, and 14 on adults or all age groups [71].

Most papers used a recommended CHNRI time frame of 10 years (n = 37), 10 had shorter, and three had longer time frames. The natural evolution of the originally proposed CHNRI method through its implementations is exemplified best in the modification of the criteria used for prioritisation by the users. In fact, only one-third of the exercises retained the original five CHNRI criteria, with most modifications including both the changes in the number and the choice of specific criteria. Five criteria were most frequently used (n = 28), followed by four or three (n = 6), but about a third of papers increased the number of criteria (n = 16), where one exercise used 13 of them. The five ‘standard’ CHNRI criteria were the most frequently used (from n = 43 for the ‘equity’ criterion to n = 35 for the ‘effectiveness’ criterion), but the most frequent new criteria were ‘feasibility’ (n = 11), ‘acceptability’ (n = 11), ‘low cost’ (n = 11), ‘sustainability’ (n = 11), ‘relevance’ (n = 6), and ‘applicability’ (n = 6). This showed the flexibility of the CHNRI method, where adjustments of the process to the needs of each specific exercise are encouraged [71].

Several broad messages emerged for health research policy across these 50 CHNRI exercises. When a health issue was poorly understood – its population burden, risk factors, or potential interventions – epidemiological research was usually the top priority, reflecting the need to define the problem’s ‘architecture’. For most contemporary issues, however, where burden and risk factors are known and interventions exist but are underused, delivery research dominated – including health systems, policy, operations, and implementation research – particularly in LMICs. This emphasis was reinforced by the short timeframes (often 10 years) and urgency of reducing child mortality. In contrast, longer horizons (20–30 years) or conditions lacking effective interventions shifted priorities toward translational or discovery research. Translational questions scored highly when existing interventions required simple adaptations (*e.g.* heat-stable vaccines), while discovery research was prioritised for poorly understood conditions such as dementia. Time horizons adopted by funding agencies strongly influence such prioritisation [71].

### The apparent advantages of the CHNRI method

The first review of the conducted CHNRI exercises implied that the popularity of the method resulted from several key advances. First, the method is systematic, providing a framework to handle an unlimited range of research questions across all categories of health research. Second, it is transparent, with clearly defined context and criteria, and with all inputs documented in numerical data sets, ensuring replicability. Third, it is democratic, relying on a crowdsourcing approach for both submitting and scoring questions. This prevents any individual from exerting undue influence, as results reflect the collective judgment of a broad group of experts. Fourth, the process is inclusive, fostering ownership by engaging donors, researchers, and other stakeholders. Donors define context and criteria, researchers contribute and score questions, and stakeholders can assign weights to criteria. Fifth, it is flexible, as it can be easily adapted to diverse contexts by modifying criteria or components. Sixth, it is simple, requiring only ‘yes’/‘no’ inputs and producing intuitive 0–100% scores that reflect collective optimism. Seventh, the results are transparent, replicable, and amenable to quick and easy validation, which does not even require complex statistical methods, although they can be added. Eighth, the method is quite inexpensive to conduct, given the usefulness of its outputs. Finally, its structured and objective outputs are publishable in scientific journals and easy to disseminate globally [71].

### Further notable examples of implementation

Several high-profile priority setting exercises were published in respected scientific journals in 2016. ‘The Lancet Global Health’ published the exercises by Dua and colleagues on child development [72] and Kennedy and colleagues on the quality of care for women [73]. Meanwhile, ‘The Lancet Neurology’ published a highly impactful paper by Shah and colleagues on setting global research priorities in dementia research [74]. Also, Nagata and colleagues published their CHNRI-based exercise to set research priorities in the field of adolescent health in the ‘Journal of Adolescent Health’ [75]. Most of these exercises were supported or led by WHO-based experts. By the end of 2016, the number of the published exercises using the CHNRI method to set research priorities has surpassed 60, averaging one published exercise for every two months over the previous decade since the method has been first introduced.

## YEARS 2017–2019

### Further notable examples of global-level implementation

Sir Aziz Sheikh and the WHO set research priorities for medication safety in the context of the WHO’s Third Global Patient Safety Challenge: Medication Without Harm. The exercise included world-leading researchers in patient and medication safety and a pool of experts from the WHO Global Patient Safety Network, and it generated research priorities separately for high-income countries (HIC) and LMICs [76]. Also, the NIHR Global Health Research Unit on Global Surgery (GlobalSurg) established research priorities within the domains of access to surgery, cancer, perioperative care, research methods, acute care surgery, and communicable disease, with two-thirds of the scoring coming from the clinicians, patients, and other stakeholders based in LMICs [77].

### Further regional- and national-level implementations of the CHNRI method

A leading example of the regional implementation was the one by the NIHR Global Health Research Unit in Respiratory Health (RESPIRE), a collaboration between the University of Edinburgh, UK, and four countries in South-East Asia (Bangladesh, Pakistan, India, and Malaysia) which conducted an internal process of identifying research priorities for respiratory health issues in this region [78].

In this period, the CHNRI method also found its implementation within Europe. The WHO European office conducted the CHNRI-based exercise to define the tuberculosis research agenda, as nine countries in the WHO EURO region were in the top 30 countries with the highest burden globally of multidrug-resistant tuberculosis [79].

The period between 2017–2019 saw some landmark examples of national-level implementation that truly surpassed all previous efforts in their ambition, size, and scope. The two papers that described a national-level exercise in India, led by Narendra Arora and the Indian Council of Medical Research (ICMR) and the INCLEN, and assisted methodologically by Kerri Wazny, stand out as possibly the best examples to date of how the method can be applied at the national level to assist the government in setting health research priorities.

In India, Arora and colleagues undertook a nationwide exercise through which they managed to engage faculty from 256 institutions to identify research priorities in the maternal, newborn, and child health and nutrition themes for 2016–25 [80]. The context of the exercise was defined by a national steering group and guided by four thematic research subcommittees. The former obtained research ideas from 498 Indian experts and then engaged 893 experts in scoring against five pre-defined criteria – ‘answerability’, ‘relevance’, ‘equity’, ‘investment’, and ‘innovation’. They also organised a larger reference group to assign weights to criteria. The Indian team produced a ranked list of priorities for each of the four themes (maternal, newborn, and child health and nutrition) at national and three subnational levels. They noticed that research priorities differed between regions, and that delivery research ideas that included implementation research contributed about 70% to the top ten research ideas for all four themes [80]. A follow-up paper to this exercise that focussed only on child health was then published by Wazny and colleagues [81], where 90 experts contributed 596 suggestions consolidated into 101 research ideas, which were then scored by 233 Indian experts.

Another landmark example of national-level implementation can be credited to the PhD work of Parisa Mansoori, the third doctoral student who chose the CHNRI method as her topic of interest. Working within a five-member management team and the Iranian CHNRI Health Research Priority Setting Group, she led a national-level CHNRI exercise for Iran which engaged 48 prominent Iranian academic leaders, a group of research funders and policymakers, and 68 stakeholders from the wider society [82]. They scored 128 proposed research ideas independently using a set of five criteria: ‘feasibility’, ‘impact on health’, ‘impact on economy’, ‘capacity building’, and ‘equity [82].

## YEARS 2020–2023

### The implementation of the CHNRI method during the COVID-19 pandemic

The CHNRI method has demonstrated its utility in the years 2020–2023 during the COVID-19 pandemic. This context was entirely different from the previous CHNRI-based exercises because it required urgency, while decisions often needed to be made in the context that lacked important information. However, there were several excellent examples where the CHNRI method assisted researchers, stakeholders and policymakers alike.

The International Society of Global Health (ISoGH) identified research priorities to reduce the impact of COVID-19 in LMICs by considering 192 research questions that were scored by 52 experts. Among the top 10 research priorities, research questions related to vaccination were prominent, joined by the questions on effective strategies to manage COVID-19 globally and in LMICs, integrating health care for COVID-19 with other essential health services in LMICs, and assessing COVID-19 patients’ needs in rural areas. The ISoGH called on the funders of health research in LMICs to consider the urgency and priority of this research during the COVID-19 pandemic and support studies that could make a positive difference for their populations [83]. Following this effort, an international collaboration called the ‘COVID-19 Research Prioritization Group on Maternal, Newborn, Child and Adolescent Health (MNCAH)’ used the CHNRI method to identify major research gaps to mitigate the direct and indirect effects of the COVID-19 pandemic on MNCAH [84].

Another collaboration, the ‘International COVID-19 Airways Diseases Group’, conducted a CHNRI exercise to identify research priorities to better understand long-term sequelae of COVID-19 in patients with pre-existing and new-onset airways disease. A list of 98 research topics was scored by 48 experts, while patients with pre-existing or post-COVID-19 airways disease contributed by weighing selected criteria. The highest-ranked research ideas focussed on the investigation of the relationship between prognostic scores at hospital admission and morbidity after hospital discharge in patients with and without pre-existing airways disease. High priority was also assigned to comparisons of the prevalence and severity of post-COVID-19 fatigue, sarcopenia, anxiety, depression, and risk of future cardiovascular complications in patients with and without pre-existing airways disease. Presenting their findings in ‘The Lancet Respiratory Medicine’ [85], the authors suggested adopting the same prioritisation process to other, non-respiratory aspects of long COVID.

Francis-Oliviero and colleagues used the CHNRI method to set research priorities to increase vaccination coverage in Europe through the European Union’s joint action on vaccination. They prioritised approaches for generating and synthesising evidence to support policies and strategies aiming at increasing vaccine coverage. They initially focussed on four pre-selected pilot vaccines – pertussis, measles-containing combination vaccines, influenza and HPV – but aim to continue and identify vaccination research priorities regarding all vaccines used in the EU, including COVID-19 vaccines [86].

### Substantial methodological improvements through the activities of the ISoGH

Peige Song is the fourth among my former PhD students who contributed to the advancement of the CHNRI method. She was only introduced to the method towards the end of her studies, but made significant methodological contributions during her post-doctoral years, working with Yajie Zhu. She noticed that the originally proposed CHNRI process could use more sophisticated statistical methods to analyse the input from the scorers, thus making it more robust and statistically rigorous. As she was later also elected to the role of the vice-president of ISoGH, the majority of methodological progress on the CHNRI method during the period 2020–2023 was achieved through the activities of the ISoGH and its own priority-setting exercises.

First, Song noticed that the *post-hoc* analysis of the experts’ scores could address uncertainty and compute confidence intervals of each ‘research priority score’. She and Zhu then suggested using bootstrapping to compute the 95% confidence intervals. They further noticed that the agreement statistics could be methodologically strengthened, exposing the most and the least controversial research ideas. Therefore, they improved the originally proposed CHNRI’s average expert agreement (AEA) score using information theory by defining this metric as the exponential of the negative entropy. Entropy is a widely used information criterion to quantify uncertainty; in the case of CHNRI scores, higher entropy implies greater uncertainty and less agreement. In contrast to the original AEA, which only used the most frequent score class for calculation, the improved AEA metric considered all score classes simultaneously, offering a more informative, robust, and theoretically guided interpretation [17].

Another need that is often present when the CHNRI papers are reviewed is demonstrating that there is no sub-structuring among the scorers that could bias the overall scores. One need is to analyse experts' scores to identify any ‘clusters’ of experts who scored very similarly. Another need is to explore how large such clusters may be, and how they affect the overall scores, because this could control for potential bias. In recent CHNRI-based exercises, Song and Zhu addressed this problem by assessing the diversity of expert scoring through hierarchical clustering on both the overall scores and those within each criterion [17,87].

### Further notable examples of global-level implementation

Among the many notable examples of the global-level implementation from this period, I will reflect on the three studies. Paskins and colleagues, on behalf of the Musculoskeletal Disorders Research Advisory Group *vs*. Arthritis, identified research priorities to reduce the impact of musculoskeletal disorders, which they published in ‘The Lancet Rheumatology’ in 2022. They engaged 213 people in the first survey and 285 people in the second, representing clinicians, researchers, and people with musculoskeletal disorders. Key priorities included developing and testing new treatments, improving treatment targeting, early diagnosis, prevention, and advancing understanding and management of pain, with emphasis on underlying mechanisms. They also issued a call to action for researchers and funders to address these priorities. [88]

Adeloye and colleagues conducted a CHNRI-based exercise within the NIHR RESPIRE collaboration that involved many globally recognised experts in chronic obstructive pulmonary disease (COPD). They identified research priorities that need to be addressed in the next 10 years to substantially reduce the global impact of COPD based on 230 research ideas scored by 34 experts. Of the top 20 overall research priorities, six were focussed on feasible and cost-effective pulmonary rehabilitation delivery and access, particularly in primary/community care and low-resource settings, while a further three called for research on improved screening and accurate diagnostic methods for COPD in low-resource primary care settings [89]. These studies stand out as excellent examples of global CHNRI exercises focussed on highly prevalent non-communicable diseases.

Ko and colleagues applied the CHNRI methodology to prioritise research to enable the implementation of the initiative ‘Ending Cholera: A global roadmap to 2030’. A list of research questions was derived from the Working Groups of the Global Taskforce for Cholera Control and other experts, engaging 177 experts and stakeholders and prioritising 32 research questions that consider both immediate and long-term Roadmap goals [90].

### Further notable examples of regional- and national-level implementation

By early 2020 and the beginning of the COVID-19 pandemic, the CHNRI method had already been implemented in several regions of the world. The first national- and regional-level examples of implementation were conducted and published for African countries (starting in 2007), then South America (starting in 2012–2013), followed by China (starting in 2014–2015), South-East Asia (starting in 2017–2018), West Asia (starting in 2018), and Europe (starting in 2019). However, in the period 2020–2023, the CHNRI method managed to make an impact in all the remaining world regions.

The first example of a regionally engaged approach in the Western Mediterranean region is the study by Mandil and colleagues from the WHO Regional Office for the Eastern Mediterranean in Cairo, Egypt. They conducted a CHNRI-based exercise to develop priorities for a regional research agenda and its implementation in Jordan and Pakistan, which was published in the ‘Eastern Mediterranean Health Journal’ in 2021 [91]. In Jordan, the CHNRI-based priority-setting exercise was aligned with the Ministry of Health’s ‘Strategic Plan and National Action Plan for Health Security’, involving 40 research experts and stakeholders in close collaboration with the Ministry itself. In Pakistan, the exercise was linked to the ‘12th National Five-Year Plan’ and the WHO’s ‘Country Cooperation Strategy’, engaging 50 stakeholders from the Ministry of National Health Services, academic and research institutions across sectors, the WHO, and other development partners. These efforts identified 30 research priorities for Jordan and 50 for Pakistan. Now, the WHO Regional Office for the Eastern Mediterranean aims to extend such exercises to other Member States in the Eastern Mediterranean Region, ensuring resources target high-priority health problems and support policy-making and sustainable development. Success will be tracked using indicators such as stakeholder satisfaction with the process, awareness and citation of priorities, changes in research funding compared to baseline, and shifts in the focus of research, including development of novel interventions [91].

Two regional-level examples from Africa that were conducted in this period also deserve mention for their ambition and an impressive expansion of the CHNRI method’s implementation in challenging settings. Leopold Ouedraogo from the WHO Regional Office for Africa in Brazzaville, Congo, and his colleagues published an entire series of the CHNRI-based exercises in the journal ‘Advances in Reproductive Sciences’ in 2021. The themes chosen for health research prioritisation included reproductive, maternal health, and ageing; mHealth and innovative strategies in sexual and reproductive health and rights; cervical cancer prevalence, prevention and treatment; gender-based violence; preventing unsafe abortions; sexual and reproductive health and rights, including a separate exercise for humanitarian settings [92-97].

Alobo and colleagues published another impressive CHNRI exercise focussed on the African region in the journal ‘AAS Open Research’ in 2021. Research priorities in maternal and neonatal health were established by engaging over 900 experts across Africa. From the 609 proposed ideas, 46 were prioritised. The leading ones focussed on improving the identification and diagnosis of high-risk mothers and newborns; also, enhancing access to treatment through incentives for attracting and retaining skilled health workers in rural areas; strengthening emergency transport; and assessing health system readiness; and increasing uptake of proven interventions such as kangaroo mother care. Emphasis was placed on expanding access to quality care at the lowest delivery levels and ensuring community participation in interventions [98].

Finally, the CHNRI method started finding its applications even in the high-income regions such as North America and Australia, although these exercises are focussed on entirely different themes in comparison to those in LMICs. An example from Canada includes the study from Cheung and Du Mont, who built a Canadian research agenda to address gender-based violence against trans people and presented their work at the University of Toronto in 2021, publishing it later in ‘Health Research Policy and Systems’ journal [99]. One example from the USA is the study by Kelly and colleagues, who identified research priorities for safer in-person school for children with medical complexity during the COVID-19 pandemic, which they published in the journal ‘Pediatrics’ in 2022 [100]. An example from Australia is the study by Beck and colleagues published in the ‘Journal of Transport and Health’ in 2022, which reported on the development of active transport research priorities for Australia [101]. The themes of these three exercises reflect that the topics in need of research prioritisation differ between LMICs and HIC, but the CHNRI method can still be useful in both regions of the world in addressing a multitude of challenges.

## YEARS 2024–2025

Today, nearly two decades after the CHNRI method was introduced, more than 200 exercises have been published in the world literature that were based on the standard, or the modified version. The method has been widely adopted by major international organisations such as the WHO and UNICEF as their suggested approach to setting research priorities. It has been used by many international organisations, professional societies, and national governments to set health research priorities, spanning a wide spectrum of topics and publishing the results in the leading medical journals.

### Further notable examples of global, regional, and national-level implementation

The period of 2024–25 brought about some of the best-designed and most rigorously conducted CHNRI exercises at the global level, many of them under the WHO’s coordination. Bertagnolio and colleagues published WHO’s global research priorities for the highly topical issue of antimicrobial resistance in human health in ‘The Lancet Microbe’ journal in 2024 [102]. Gottlieb and colleagues published the WHO’s global research priorities for sexually transmitted infections in ‘The Lancet Infectious Diseases’ in 2024 [103]. On behalf of the WHO, Brennan-Wilson and colleagues set global research priorities for engaging men and boys in sexual and reproductive health and rights, and doing so in a way that challenges harmful masculinities, which was published in ‘The Lancet Global Health’ in 2024 [104]. Lohan and colleagues coordinated the WHO’s global research priority-setting exercise on the sexual and reproductive health and rights of young adolescents, which was published in ‘The Lancet Child and Adolescent Health’ in 2025 [105]. Williams and colleagues coordinated the WHO’s research agenda for ending preventable maternal deaths from postpartum haemorrhage, published in the journal ‘BMJ Global Health’ in 2024 [106].

Wood and colleagues prepared a background paper for the UNICEF in 2025 focussed on identifying research priorities for the effective inclusion of children with disabilities in LMICs [107]. The UNICEF and the WHO also co-published an extensive report on global research priorities at the intersections between violence against children and violence against women [108]. Several other exercises were commissioned by the WHO to set research priorities in different areas of global health, including the child health research agenda, the validation of the use of traditional medicine for healthy ageing, and others. Therefore, this trend of highly visible and impactful CHNRI exercises should be expected to continue in the coming years, both at the global and the regional levels.

Special initiatives and international collaborations are being formed in the meantime, too, which address specific priorities in global health. Recently, the Climate Change and Health Impact Research Priorities Group published a preprint of their research priority setting exercise to both understand and address the impact of climate change on the health of women and children in LMICs [109]. The ISoGH published global research priorities for pandemic preparedness based on both human and AI input [17], while its most recent exercise, which is awaiting publication, addressed research priorities for the use of data science and artificial intelligence in global health [87]. D'Mello-Guyett and colleagues identified research priorities for water, sanitation, and hygiene in humanitarian crises [110], while Corboz and colleagues published a global shared research agenda on violence against women in LMICs [111]. Uysal and colleagues developed a global research agenda for adolescent pregnancies, addressing social norms for adolescent timing and spacing of pregnancy in LMICs [112]

An example of progress at the regional level is the work by Lambach and colleagues developed on behalf of the WHO SEARO region, which reports on the WHO’s Immunisation and Vaccines-Related Implementation Research Advisory Committee meeting in 2024, where the CHNRI exercise was used to set research priorities in immunisation programme strengthening for South-East Asia [113].

There have also been some notable examples of CHNRI exercise successfully conducted during this period in very challenging settings at the national level, too. El Baz and colleagues and the Afghanistan Health Systems Research Prioritization Collaborators completed the first research priority setting exercise for Afghanistan [114], while Majumdar and colleagues and the Afghanistan Maternal, Newborn, and Child Health Research Prioritization Collaborators established research priorities for maternal, newborn and child health, sexual and reproductive health, and nutrition [115]. Both papers were published in the journal ‘BMJ Glob Health’ in 2025. Sayeed and colleagues completed stakeholder-led research priorities for advancing sexual and reproductive health and rights in Bangladesh [116], Motevalian and colleagues identified health research priorities in Pakistan [117], while Durão and colleagues set research priorities for newborn and child health in Malawi, Nigeria, and South Africa [118].

### The rise of AI and its inclusion in the CHNRI method

In 2024, a major, unforeseen change affected the development and implementation of the CHNRI method. Originally, it was envisaged as the method that assigns scores and ranks to research ideas that were generated, evaluated and prioritised by humans. However, the advent of large language models such as ChatGPT, DeepSeek, Claude, Grok, and others has changed the context entirely. AI can now also generate research ideas, evaluate them using different criteria, and propose research priorities. To the best of our knowledge, we were the first to demonstrate that ChatGPT is able not only to generate and assess research ideas, but also to write an entire research article [119]. Also, the recent research priority setting exercise on pandemic preparedness, which Peige Song and I coordinated for the ISoGH, was the first CHNRI-based exercise that compared research priorities based on human collective opinion with those generated by ChatGPT, which was trained on human collective knowledge. The results showed both striking similarities and interesting differences [17]. Thus, we envisage that, in the future, CHNRI-based processes may assist in studying how does human collective opinion on many ideas differ from the suggestions generated by AI. The CHNRI method may become one of the first examples in which using both human and AI-generated views will, in fact, strengthen the confidence in their conclusions wherever they are mutually supportive. Such comparisons may also point to interesting new findings wherever they start to differ, enabling further progress in this new field of science that will compare human-generated and AI-generated priority ideas within the field of ideometrics.

In an interesting recent viewpoint, Garry and colleagues discussed the potential role of AI in research priority setting exercises [120]. They highlighted several imperfections and limitations of human-led research priority-setting exercises, as well as AI-based tools. Similar to the conclusions from Song and colleagues [17,87], they also realised the potential in comparing both approaches to learn from their differences and to provide more assurance to funders and policymakers wherever the results overlap. They also encouraged research that could identify the extent to which AI can replicate conventional research priority-setting exercises [120].

### Ethical considerations and reporting guidelines for research priority setting

An appreciable effort was made recently by Joseph Millum in 2024 to consider the ethical aspects of priority-setting exercises in health research [121]. He reviewed the literature on the ethics of health research priority setting, comparing different methods that have already been developed and identifying themes in the current discussions about ethics and priority setting [121]. His findings are expected to inform guidance from the WHO on how to incorporate ethics into the processes of health research priority setting, which could further strengthen them regardless of the methods used.

Another progress that also deserves a mention is the proposal of a “REporting guideline for PRIority SEtting of health research” (REPRISE) [122]. This guideline was published by Tong and colleagues, who rightly suggested that ‘ensuring transparency of the priority setting process can strengthen legitimacy and credibility for influencing the research agenda’. They checked 21 556 records and included 26 sources for the candidate REPRISE framework and 455 primary research studies. Their REPRISE guideline contains 31 reporting items that cover 10 domains of typically used approaches in health research priority setting, including the CHNRI method. The REPRISE guideline is a welcome addition to this field, expected to facilitate comprehensive reporting of research priority setting exercises [122].

### The first evaluation of the impact of the CHNRI method’s implementation

Over the past two decades, the CHNRI method has achieved global recognition, uptake and implementation by numerous international organisations, governments and groups of prominent researchers. However, to demonstrate the value of the method, and of the emerging scientific field of ideometrics, its real-world impact will need to be scientifically evaluated against the alternative – i.e., the scenario that would evolve in the absence of the CHNRI-based or ideometrics-based prioritisation process. A rare attempt to scientifically evaluate the effect of the CHNRI method’s implementation has been published by Gupta and colleagues from the WHO’s Department of Maternal, Child, Adolescent Health and Ageing [123]. In a field of global newborn health research, which is relatively small and highly specific, and thus suitable for such analysis, the authors used the list of the top 20 priorities identified by the same department in 2014 [51]. They then conducted searches of the databases with the world’s scientific literature, clinical trial registries, and funder websites for the period between July 2014 and May 2022, looking for any studies of adequate design that addressed one of the top 20 research priorities. They then assessed the level of uptake of each identified priority based on a set of predefined criteria. They found that the uptake of the 20 research priorities identified in 2014 was high for 8 priorities (40%), moderate for 11 priorities (55%), and low for 1 priority (5%). They concluded that a fair amount of research has been conducted to address the most pressing global research priorities in newborn health identified in 2014, and that funders and researchers alike should reflect on and think about addressing less researched areas [123].

## BRAIN’S PERCEPTION OF IDEAS AS AN UNDERAPPRECIATED HUMAN SENSE

I recently made further theoretical progress, based on my experience with the CHNRI method, that could give rise to a new scientific field. Namely, based on nearly two decades of implementing the CHNRI method with many groups and working through more than 20 000 research ideas, I proposed that humans may have an underappreciated sense – the ‘perception of ideas’ – with a human brain being the primary sensory organ [124]. From the brain’s sensory perspective, ideas are competing possibilities of purposeful activities that, if followed, are expected to result in an alternative version of the future. I noticed that the exposure of the researchers involved in the CHNRI process to proposed new research ideas can instigate physical, physiological and psychological responses, ranging from sitting up, increased psychomotor activity, apparent verbal enthusiasm and excitement, to feeling concerned or even threatened. The judgment on which ideas to prioritise is affected by education, experience, and cognitive abilities; it can likely be sharpened through increased quantity of valuable information about the context, while misinformation can negatively affect the brain’s prioritisation of ideas and decision-making. Based on internal and external stimuli, our brains continuously generate, evaluate, and prioritise ideas. Pursuing the prioritised ideas drives human activity, requiring prioritisation between short-, mid-, and long-term investment of energy and time [124].

## THE CONTRIBUTION OF THE CHNRI METHOD TO THE EMERGING FIELD OF IDEOMETRICS

This new angle on understanding of the brain's sensory role in continuously assessing many competing ideas may suggest that prioritising ideas for health research using the CHNRI method is merely a highly specific case of a much more general process. This process could generally be called ‘ideometrics’, as it essentially attempts to ‘measure ideas’ – as we implied in the title of the first book on the CHNRI method in 2022 [2]. It is, therefore, analogous to the examples of scientometrics, econometrics, informetrics, psychometrics, and several other fields. In all those examples, the respective scientific fields were either established or revolutionised by introducing novel approaches to successfully measure the subjects of interest and then analyse their quantitative properties.

As an emerging field of science, ideometrics is fundamentally based on the human brain’s ability to generate, evaluate, and prioritise ideas on how to address just about any situation. In doing so, as described in my ‘sense of ideas’ work [124], it uses criteria that typically belong to three large groups – a motivational/emotional (an idea’s attractiveness), an operational/rational (an idea’s feasibility), and an outcome-related perspective (an idea’s impact). The brain’s eventual prioritisation of ideas will depend on the value that it assigns to information that it has about a context. As explained recently my other paper [67], information that the brain will consider valuable will have some inherent traits: relevance (i.e., influences the perception of the related beliefs), credibility (i.e., needs to be trusted), and leverage (i.e., influences ideas and decisions decisively). Where all three criteria align, information assumes high value and serves in revising prioritised ideas.

As an emerging quantitative field of science, ideometrics should not remain limited to health research. Instead, it should continue developing and applying methods to generate, evaluate and prioritise ideas for just about any purpose, and revise the ones already prioritised. Initially, it could be applied in many areas of science outside of health to set research priorities – *e.g.* in physics, chemistry, and biology, computer science, social and political sciences, materials sciences, and many others. In parallel, it could be used to address the most pressing challenges in the public sector, *i.e.* in governments’ decision-making, using crowdsourced opinion from many experts. It could also be applied in the private sector to crowdsource the best ideas from leading managers about the future development of their companies. Ultimately, it could be applied by a country’s entire population, or its sizeable proportion, through the collection of people’s voluntary input of ideas, knowledge and/or opinions through the most appropriate means, to transform society for the better. The mechanism towards this goal would be to simply follow those ideas that are more likely to bring positive change. This could be achieved based on an informed understanding of both the context and the key criteria that could make ideas more likely to succeed in reaching the society’s goals within the specific context. People could gradually learn to jointly prioritise and follow the ideas that are most likely to bring prosperity to their society, while also protecting themselves from those ideas that are unlikely to do so, based on a transparent process that should ensure better understanding of the choices that are presented.

Thoughts of a possible emerging field of ‘ideometrics’ and the position of the CHNRI method within this scientific field led me to explore other methods and approaches that perform the CHNRI method’s role in other areas of human activity, i.e. to generate, evaluate, and prioritise ideas. I made a very preliminary list, which is neither complete nor comprehensive – it is just a start. The purpose of presenting it here is to demonstrate a surprising finding: humans have been using dozens of methods and tools throughout their entire history to generate, evaluate, and prioritise ideas. The list shows about 70 such methods and contains key references describing each approach [125–205] (**Table 1**). Those methods have been applied in vastly different and diverse areas of human activity; some were primarily used to generate, some to evaluate, and some to prioritise ideas; some of them address more than one of those needs. The CHNRI method is among the rare ones to provide a solution for addressing all three of the steps of the ‘ideometrics’ process (**Table 1**).

**Table 1:** The landscape of approaches, methods and tools that have been historically used to generate, evaluate and prioritise ideas.

Idea generation methods (approaches used to produce novel ideas, concepts, or hypotheses)	Individual and cognitive techniques	Stream of consciousness and freewriting (William James, 1890 [125]; Peter Elbow, 1973 [126])
		Theory of Inventive Problem Solving – TRIZ (Genrich Altshuller, 1946 [127])
		Morphological analysis (Fritz Zwicky, 1957 [128])
		Lateral thinking (Edward de Bono, 1967 [129])
		SCAMPER technique (Robert F. Eberle, 1971 [130])
		Mind mapping (Tony Buzan, 1974, 1993 [131,132])
		Heuristics and biases framework (Amos Tversky and Daniel Kahneman, 1974 [133])
		Six Thinking Hats (Edward de Bono, 1985 [134])
	**Group-based and social approaches**	Brainstorming (Alex F. Osborn, 1942, 1953 [135,136])
		Focus groups and in-depth interviews (Robert K. Merton, Marjorie Fiske, Patricia L. Kendall, 1956 [137])
		Delphi technique (Norman Dalkey and Olaf Helmer, RAND Corporation, 1963 [138])
		Nominal Group Technique (Andre L. Delbecq and Andrew H. Van de Ven, 1971 [139])
		World Café (Juanita Brown and David Isaacs, 1995 [140])
		Open Space Technology (Harrison Owen, 1997 [141])
		InnoCentive (Alpheus Bingham, Aaron Schacht, and Dwayne Spradlin, 1998, 2011 [142])
		James Lind Alliance (Nick Partridge and John Scadding, 2004 [143], with Iain Chalmers)
		Child Health and Nutrition Research Initiative – the CHNRI method (Igor Rudan, 2006, 2008 [1,6])
		IdeaScale (Vivek Bhaskaran and Rob Hoehn, 2009 [144])
	**Design and innovation frameworks**	Human-Centered Design (John E. Arnold, 1958 [145]; Donald A. Norman, 1988 [146]; IDEO.org, 2009 [147])
		Design Thinking (Herbert Simon, 1969 [148]; Tim Brown, 2008 [149])
		Agile ideation sprints (Ken Schwaber and Jeff Sutherland, 1995 [150])
		Hackathons (John Gage and OpenBSD, 1999 [151])
		Lean Startup Methodology (Eric Ries, 2011 [152])
	**Computational and AI-driven methods**	Genetic algorithms and evolutionary computation (John Holland, 1975 [153])
		Automated hypothesis generation (Don R. Swanson, 1986 [154])
		Generative adversarial networks for idea synthesis (Ian Goodfellow, 2014 [155])
		Large language models for ideation support (Ashish Vaswani et al., 2017 [156], OpenAI, 2019 [157])
**Idea evaluation methods (techniques to judge the quality, feasibility, novelty, or value of proposed ideas)**	**Expert-based evaluation**	Peer review (Henry Oldenburg, 1665 [158])
		Modified Delphi for scoring (Norman Dalkey and Olaf Helmer, RAND, 1963 [138])
		Expert panels and consensus conferences (National Institutes of Health, 1977, 1990 [159])
		Analytical Hierarchy Process (Thomas L. Saaty, 1977,1980 [160,161])
	**Quantitative assessment metrics**	Cost-effectiveness and cost-benefit analysis (Abbé de Saint-Pierre, 1708 [162]; Ezra J. Mishan, 1971 [163])
		Net present value (NPV) and internal rate of return (IRR) (Irving Fisher, 1907 [164]; Joel Dean, 1951 [165])
		Patent metrics (US Patent & TM Office, 1947; Adam Jaffe, Manuel Trajtenberg, Bronwyn Hall, 1993 [166])
		Bibliometric and scientometric indices (Eugene Garfield, 1955 [167] and 1972 [168]; Jorge Hirsch, 2005 [169])
		Technology Readiness Levels (Stanley Sadin and NASA 1970s [170], John C. Mankins 1995 [171])
	**Scoring models and criteria-based frameworks**	Multi-criteria decision analysis (MCDA) (Harold W. Kuhn and Albert W. Tucker, 1951 [172])
		SWOT analysis - Strengths, Weaknesses, Opportunities, Threats (Albert S. Humphrey, 1960s [173])
		Weighted scoring models (Stanley Zionts, 1979 [174])
		Pugh matrix – decision-matrix method (Stuart Pugh, 1980s, 1990 [175])
		Feasibility-Desirability-Viability framework (Tim Brown, 2009 [149])
	**Crowd-based assessment**	Wisdom of the crowd techniques (Francis Galton, 1907 [176])
		Prediction markets (Robin Hanson, 1980s, 1990 [177])
		James Lind Alliance (Nick Partridge and John Scadding, 2004 [143], with Iain Chalmers)
		Child Health and Nutrition Research Initiative (CHNRI) method (Igor Rudan, 2006, 2008 [1,6])
		Social media engagement metrics as proxies for idea traction (Jason Priem, 2010 [178])
	**Scientific and philosophical validity tests**	Logical consistency and deductive reasoning (Aristotle, 4th century BC [179,180])
		Empirical testability, replicability and falsifiability (Francis Bacon, 1620 [181]; Karl Popper, 1934 [182])
		Paradigm shift (Thomas Kuhn, 1962 [183])
**Idea prioritisation methods (methods to select the most promising ideas for action, investment, or further study**	**Structured decisionmaking frameworks**	Paired comparison methods (Louis L. Thurstone, 1927 [184])
		Multi-voting and Dot-voting (group facilitation practices, 1950s–1960s [185,186])
		Delphi with ranking rounds (Norman Dalkey and Olaf Helmer, RAND, 1963 [138])
		Nominal Group Technique with voting (Andre L. Delbecq and Andrew H. Van de Ven, 1971 [139])
		Analytic Hierarchy Process (Thomas L. Saaty, 1977,1980 [160,161])
	**Priority-setting frameworks in health and science**	RAND/UCLA Appropriateness Method (RAND Corporation and UCLA clinicians, 1980s, 2001 [187])
		Essential National Health Research framework (COHRED, 1990 [188])
		GRADE methodology with EtD (Evidence to Decision) frameworks (GRADE Working Group, 2000 [189])
		James Lind Alliance Partnerships (Nick Partridge and John Scadding, 2004 [143], with Iain Chalmers)
		Combined Approach Matrix (Abdul Ghaffar, Andres de Francisco, Stephen Matlin, 2004; [190])
		Child Health and Nutrition Research Initiative (CHNRI) method (Igor Rudan, 2006, 2008 [1,6])
	**Portfolio and pipeline management tools**	Real Options Analysis (Stewart C. Myers, 1947 [191])
		R&D portfolio matrices (Bruce Henderson and Boston Consulting Group, 1970 [192])
		Product roadmapping and prioritization grids (Robert Phaal and colleagues, 1970s to 2000s, 2004 [193])
		Stage-gate model (Robert G. Cooper, 1980s, 1990 [194])
	**AI-driven prioritisation tools**	Knowledge graphs and semantic similarity clustering (Allan M. Collins and M. Ross Quillian, 1960s [195])
		Reinforcement learning-based portfolio optimization (Richard S. Sutton and Andrew G. Barto, 1998 [196])
		Automated priority setting *via* large language models (Peige Song and Igor Rudan, ISoGH, 2024 [17])
	**Participatory and democratic prioritisation**	Cross-Cutting Philosophical and Meta-Theoretical Approaches (Paul Feyerband, 1975 [197], and others)
		Citizen juries and deliberative democracy forums (Ned Crosby, 1974 [198]; James Fishkin, 1991 [199])
		Public consultations and e-surveys with weighting (Stephen Sedley, 1985 [200])
		Participatory budgeting (Tarso Genro and Raul Pont, 1989 [201–203])
	**Approaches to prioritising ideas beyond specific techniques**	Occam’s Razor (William of Ockham, 1323-1328 [204,205])
		Bayesian inference (Thomas Bayes, 1763 [206])
		Dialectical method (Georg Wilhelm Friedrich Hegel, 1807 [207])
		Epistemic humility and pluralism (John Stuart Mill, 1859 [208])

This editorial marks a new collection of papers in the ‘Journal of Global Health’ which will attempt to characterise and theoretically advance the emerging field of ‘ideometrics’. The aim of this foundational collection will be to identify methodological approaches and define a roadmap that could lead to the prioritisation of better ideas. This would need to be based on information that has the most value, and on a scientific, quantitative approach to prioritising ideas. ‘Ideometrics’ may eventually find a novel path towards addressing the historic human challenge of scarcity, through increased efficiency and effectiveness that would arise from following ideas most likely to lead to better futures, but also avoiding those that are unlikely to achieve desired goals for individuals and populations.

## CONCLUSIONS

From its simple approach in its infancy in 2006, the CHNRI method has been continuously improved by many users over the years, increasing its scientific rigour. It gradually became apparent that the method is an attempt – albeit rudimentary – to ‘measure ideas’ using expert-sourced ‘collective optimism’ towards each idea and its components [2]. Gradually, the CHNRI method became one of the many vehicles that supported the emergence of the new quantitative field of science, which could be named ‘ideometrics’ – the science of generating, evaluating and prioritising ideas. Numerous methods that are already in use in this emerging field of science will need to continue addressing the methodological questions of the optimal number of experts to invite, a feasible number of research ideas to assess, the uncertainty of interim and final scores, agreement between scorers, and biases that could result from the composition of the scorers. They will also, inevitably, start including AI-based tools. In fact, increasing reliance on the use of AI and on statistical inference over the next several years will make both the CHNRI method and the field of ‘ideometrics’ increasingly quantitative, testable, and replicable, which should improve its standing among the many approaches that are in use to generate, evaluate and prioritise ideas today.
